# Positive Aspects of Emotional Competence in Preventing Internalizing Symptoms in Children with and without Developmental Language Disorder: A Longitudinal Approach

**DOI:** 10.1007/s10803-019-04336-y

**Published:** 2020-01-06

**Authors:** Andrea C. Samson, Neeltje P. van den Bedem, Daniel Dukes, Carolien Rieffe

**Affiliations:** 1 Institute of Special Education, University of Fribourg, St. Pierre Canisius 21, 1700 Fribourg, Switzerland; 2 Faculty of Psychology, Swiss Distance University Institute, Überlandstrasse 12, 3009 Brig, Switzerland; 3 Developmental Psychology, Leiden University, Wassenaarseweg 52, 2333AK Leiden, The Netherlands; 4 Swiss Center for Affective Sciences, University of Geneva, Chemin des Mines 9, 1202 Geneva, Switzerland; 5 NSDSK, Lutmastraat 167, 1073 GX Amsterdam, The Netherlands; 6 Department of Psychology and Human Development, Institute of Education, University College London, UCL, 25 Woburn Square, London, UK

**Keywords:** Specific language impairment, Internalizing psychopathology, Longitudinal study, Protective factors, Positive emotions, Emotion awareness

## Abstract

In order to better understand protective factors for internalizing problems, this longitudinal study examined positive emotions, emotion awareness and (non-)emotional communication skills in relation to somatic complaints and social anxiety in children with (N = 104) and without (N = 183) Developmental Language Disorder (DLD) using self-reported measures twice with a 9-month interval. Additionally, parents reported on their child’s communication problems and emotion communication at Time 1. Most importantly, since we found that increasing levels of emotion awareness related to decreases in social anxiety and somatic complaints in children with and without DLD, we conclude that children with DLD are likely to benefit from interventions aimed at improving their emotion awareness in addition to language interventions.

**Electronic supplementary material:**

The online version of this article (10.1007/s10803-019-04336-y) contains supplementary material, which is available to authorized users.

Developmental Language Disorder (DLD) is characterized by profound difficulties in acquiring and using receptive or expressive language (Bishop et al. 2017). Furthermore, individuals with DLD experience increased levels of internalizing symptoms including somatic complaints and social anxiety (St Clair et al. 2010). Very little is known about potential protective factors that may inhibit such symptoms developing in individuals with DLD (such as experiencing positive emotions and being aware of and communicating about emotions), although such factors have already been well-studied in children without DLD (Rieffe et al. 2008; Rieffe and de Rooij 2012; Zeman et al. 2002) and in individuals with other developmental disorders, including Autism Spectrum Disorder (ASD) (Cai et al. 2018; Mazefsky and White 2014). The present longitudinal study aims to extend this research by examining the contribution of these potential protective factors to the well-being of children with DLD.

## Internalizing Symptoms and Developmental Language Disorder

Approximately two children in the average classroom experience significant problems developing and using language (Norbury et al. 2016). Although problems are heterogenic, children typically experience problems in the content (semantics) and the form (phonology, morphology, and syntax) of language (Bishop et al. 2017). These problems in the structural aspects of language often also cause problems in the use of language during social interactions (i.e., pragmatics; Norbury et al. 2004). In order to be diagnosed with language impairment in line with DSM-5 criteria, these specific communication problems cannot be explained by other conditions (e.g., hearing impairment, ASD), by intellectual disability, or by a general developmental delay (APA 2013). In the DSM-IV, a significant discrepancy between the non-verbal intellectual abilities and language abilities was also a prerequisite for the diagnosis (APA 1994). Therefore, these children were referred to as having a specific language impairment (SLI) in the research literature. However, in the updated version, DSM-5, this discrepancy is no longer a prerequisite for diagnosis (APA 2013; for a discussion see Bishop et al. 2017). Recently, this group of children have been referred to as having DLD, indicating that they experience significant problems acquiring and using language from early in life, and that these problems cause severe problems in daily life functioning (Bishop et al. 2017). This term will be adopted throughout this article. The communication problems of children with DLD continue to affect development, with little evidence that the differences with their peers disappear (McKean et al. 2017; Norbury et al. 2017).

As mentioned briefly above, children and adolescents with DLD also tend to experience socio-emotional difficulties and internalizing symptoms (Redmond and Rice 1998; St Clair et al. 2010) including increased levels of somatic complaints (Gregl et al. 2014; Maggio et al. 2014; Redmond and Rice 1998; van Daal et al. 2007) and social anxiety (Beitchman et al. 2001; Wadman et al. 2011). Somatic complaints, such as headaches, stomach-aches, fatigue, or other physical ailments are not uncommon in youths (e.g., about 30% of 8- to 14-year-olds report somatic complaints; Rieffe et al. 2004), and have been linked to increased stress levels, negative emotions, and depressive or anxiety symptoms (e.g., Rieffe et al. 2004; Shanahan et al. 2015). The same is true for social anxiety, which refers to the fear of social or performance situations and is linked to avoidance, anxious anticipation, or distress in such contexts (APA 2013). Clinical levels of social anxiety are reported in 5–15% of adolescents without DLD (Heimberg et al. 2000).

Although social anxiety and somatic complaints are more common in children with DLD as a group, the severity of the language problems cannot fully explain individual differences within the DLD group. For instance, the internalizing problems of children with DLD between 7 and 16 years old were unrelated to their level of expressive and receptive language problems. Only pragmatic problems, such as the initiation of conversations, non-verbal communication, use of context, and stereotypical language use, represented a risk factor for higher levels of internalizing problems (St Clair et al. 2010). Similarly, the level of somatic complaints of 5-year-olds with DLD were unrelated to their phonological, semantic, and syntactic language problems, whereas social anxiety was related to more phonological and semantic language problems (van Daal et al. 2007). Adolescents with DLD also reported more social anxiety when they had more expressive language problems, but this relation was fully mediated by their social skills (Wadman et al. 2011). Therefore, it seems warranted to look beyond the communication problems of children with DLD and try to identify other factors that might contribute to the development of somatic complaints and social anxiety.

## Protective Factors for Internalizing Symptoms

A rich literature in children without DLD suggests that protection for developing internalizing symptoms includes, amongst others, having high levels of positive emotions, emotion awareness, and the ability to communicate about emotions. Positive emotions have the power to momentarily broaden people’s repertoires of thoughts and actions (Fredrickson and Branigan 2005), improve mental and physical health (Lyubomirsky et al. 2005), and are linked to experiencing fewer symptoms in a variety of psychopathologies, including internalizing disorders (Hechtman et al. 2013; Kashdan and Roberts 2004). Positive emotions may protect mental health since they serve as a buffer against the adverse psychological and physiological consequences of negative emotions (Fredrickson 2001; Tugade and Fredrickson 2007).

Emotion awareness, which includes being able to identify, understand and label one’s own emotions, is associated with lower levels of internalizing problems in children, adolescents and adults without DLD (Begeer et al. 2008; Rieffe and de Rooij 2012; Sendzik et al. 2017). Indeed, the ability to understand one’s own emotions is crucial to being able to regulate those emotions adaptively (Lambie and Marcel 2002). However, focusing too much on the internal arousal and bodily changes of emotions is likely to diminish the attention to environmental causes of emotions, which has been associated with higher levels of internalizing problems (Rieffe and de Rooij 2012; Rieffe et al. 2008). In this sense, being relatively unaware of the body may be protective, when the situation actually requires paying more attention to the external sources of emotions (Rieffe and de Rooij 2012).

Finally, being able to communicate, especially about one’s own feeling states, may present another potential protective factor for the development of internalizing symptoms. Communicating about emotions may help focus on the social and environmental triggers rather than on bodily reactions and would suggest that children are in tune with the social environment in which the emotion-evoking event occurs (Hess 2001). Additionally, emotion communicating enables children to express their wishes and feelings, thereby affecting their social environment or their ability to gain social support (Dunn et al. 1991).

The general language problems of children with DLD may impede their ability to learn emotional skills through social interaction with their environment (Hart et al. 2006; Salmon et al. 2016; van den Bedem et al. 2019). Moreover, when children experience difficulties expressing themselves through language, this may create misunderstandings and frustration. These problems may in turn contribute to the development of somatic complaints and social anxiety over and above the severity of the communication problems of children with DLD. While there is a growing body of research on emotions in children with DLD (Bakopoulou and Dockrell 2016; van den Bedem et al. 2018a, 2019), the impact of positive emotions, emotion awareness, and emotion communication on the development of internalizing symptoms in individuals with DLD has not been studied to date.

## The Current Study

The overall goal of this longitudinal study was to examine the contribution of potential protective factors of somatic complaints and social anxiety in children with and without DLD. Specifically, the first aim was to examine the level and development across time of somatic complaints, social anxiety, happiness (as a representative of positive emotions), and emotion awareness (including emotion understanding and bodily unawareness) in children with and without DLD. The second aim was to examine whether the level and development of happiness and emotion awareness can explain individual differences in somatic complaints and social anxiety in children with and without DLD across time. The third aim was to examine whether the communication skills (structural and pragmatic) or rather the ability to communicate about emotions were related to the severity of somatic complaints and social anxiety. Furthermore, we explored whether the contribution of these factors was comparable in children with and without DLD. Because children with DLD experience more difficulties developing their emotional skills (Bakopoulou and Dockrell 2016; van den Bedem et al. 2019), these factors may have a stronger impact on the development of somatic complaints and social anxiety than in children without DLD.

## Methods

### Participants

The current study is part of a larger longitudinal study on children with and without communication problems (Rieffe et al. 2014; Theunissen et al. 2011; Van den Bedem et al. 2018b). In the current study, 104 Dutch children and adolescents with DLD and 183 without DLD participated. They were between 9 and 16 years old with a mean age of 12 years (Table 1). Participants with DLD were included when they had received a formal diagnosis of DLD in line with the DSM-IV criteria for language impairment, which is provided in the Netherlands when receptive or expressive language problems are at least 2 SD below the mean on a general language measure, or 1.5 SD below the mean on two out of four language areas (i.e. auditory working memory, speech production, syntax, and semantics). Additionally, these problems should persist after 6 months of speech and language therapy.

**Table 1:** Characteristics of participants at Time 1

	Without DLD	With DLD
Number of children—*n*	183	104
Male	76 (41.5%)	54 (51.9%)
Female	107 (58.5%)	50 (48.1%)
Mean Age in years (*SD*)	12.3 (1.4)	12.2 (1.9)
Age range in years, months	9.8–15.4	9.2–16.3
Performance IQ***	107.2 (17.2)	93.8 (12.5)
Range performance IQ	78–140	70–140
Neighborhood SES***	.72 (.95)	.06 (1.08)
Range neighborhood SES	− 2.10 to 2.44	− 4.19 to 2.50

The neighborhood SES is the mean level of education, occupation, and income of all adults in a neighborhood as compared to all other neighborhoods in the Netherlands (*M *= 0.28, *SD *= 1.09, Range − 6.8 to 3.1), ***p < .001

Most of the children with DLD were recruited through specialized schools for children with communication problems (73%), where children are educated in smaller classes by specialized teachers and often receive speech and language therapy during school hours. The other children with DLD were recruited through organizations providing support for children with DLD in mainstream education. These children and their teachers receive regular support by counsellors and children often receive speech and language therapy outside of school.

Children without DLD were recruited through mainstream schools for primary and secondary education. They were included in the control group when they had no diagnosis as indicated by their parents, had no clinical levels of language problems as measured with two subtests of the CELF (Kort et al. 2008) and had performance intelligence (PIQ) in the normal range as measured with two subtests of the WISC (see “Materials” section).

Children with and without DLD were comparable in age and gender distribution (Age: *t* (166.94) = .30, *p *= .767; Gender: *Χ*^*2*^ (1) = 2.89, *p *= .109). However, children without DLD had higher PIQ than children with DLD (*t* (260, 98) = 7.51, *p *< .001). Additionally, children without DLD lived in neighborhoods with higher socio-economic status (SES) than the children with DLD (*t* (285) = 5.30, *p *< .001). Therefore, the analyses were controlled for PIQ and SES.

## Materials

The present study used self-report measures for the internalizing problems (social anxiety and somatic complaints), emotion awareness and happiness, because these introspective topics are best judged by children themselves (Lambie and Marcel 2002). Additionally, parents reported on their child’s structural and pragmatic language ability as well as the ability to communicate about emotions. The (emotion) communication problems a child may experience in social interactions, may be best judged by the parent.

The Somatic Complaints List (Jellesma et al. 2007) assesses how often children experience bodily complaints, such as fatigue or stomach aches. Children rated whether they experienced these complaints never (1), sometimes (2), or often (3). The internal consistency of the somatic complaints list is good (Jellesma et al. 2007; Rieffe et al. 2004), as it was for children with and without DLD in the present study (*α *> .80; Table 2). Mean scores were calculated.

**Table 2:** Psychometric properties of the questionnaires

	Range	N items	α Time 1		Means (*SD*)	
			Without DLD	With DLD	Without DLD	With DLD
					183	104
Somatic complaints	1–3	11	.83	.86	1.48 (.31)	1.60 (.38)
Social anxiety	1–3	18	.87	.90	1.61 (.35)	1.72 (.40)
Happiness	1–3	5	.90	.85	2.81 (.30)	2.78 (.31)
Emotion understanding	1–3	7	.72	.78	2.40 (.34)	2.38 (.39)
Unawareness bodily symptoms	1–3	4	.72	.62	1.95 (.48)	2.17 (.47)
Communication problems N (% of total diagnostic group)					142 (77.6%)	88 (84.6%)
General***	53–157	56	.87	.91	73.13 (14.73)	115.40 (13.45)
Pragmatic***	24–78	28	.82	.85	35.86 (7.86)	54.86 (7.45)
Speech	8–24	7		.73		16.07 (3.48)
Syntax	7–20	7		.60		15.31 (2.43)
Semantic	5–18	7		.70		14.19 (1.76)
Coherence	6–20	7		.79		14.97 (2.37)
Emotion communication problems N					151 (82.5%)	87 (83.7%)
Emotion communication Problems***	1–4	14	.91	.91	1.42 (.42)	2.02 (.58)

Only the group differences for communication problems were examined; Speech, syntax, semantic and coherence problems are only examined in children with DLD because these scales are unreliable in children without DLD

*α* Cronbach’s alpha

***p < .001

The Social Anxiety Scale for Children—revised (SASC-R) (La Greca and Stone 1998) assesses how frequently (almost never (1), sometimes (2), or often (3)) children are afraid of negative evaluations, or experience stress of social situations and avoid them. This widely used questionnaire has good internal consistency (La Greca and Stone 1998), which was confirmed in for children with and without DLD in the present study (*α * > .85; Table 2).

Happiness was measured with the positive emotion labels of the Mood Questionnaire (Rieffe et al. 2004). Children indicated how often (almost never (1), sometimes (2), or often (3)) they felt this emotion during the previous 4 weeks. Internal consistency in both groups was high (*α *> .85; Table 2).

Emotion awareness was measured with two sub-scales of the Emotion Awareness Questionnaire (Rieffe et al. 2008). Emotion understanding measures the capacity of children to differentiate between their own negative emotions and to understand what caused their emotions (e.g. ‘I am often confused about how I feel’ [reversed scored]). Bodily awareness measures how much children notice their bodily reactions of emotions, such as feeling weak when being sad. Children report if they never (1), sometimes (2), or often (3) feel something in their body when they are emotional (reversed scored). Both scales have sufficient internal consistency (*α *= .64–.77) and good construct and external validity (Rieffe et al. 2008). In the present study, emotion understanding had acceptable reliability in both groups (*α *> .72), but bodily awareness was low for children with DLD (*α *= .53). After deleting one item with a negation in the question, which may have been confusing for children with DLD, the internal consistency improved (*α *= .62). In children without DLD, internal consistency was satisfactory with (*α *= .74) or without (*α *= .72) the item.

Performance IQ (PIQ) was measured with two non-verbal subtests of the WISC (Block Design and Picture Arrangement; Kort et al. 2005), which give a good indication of the PIQ of a child (Theunissen et al. 2011). PIQ scores of the children with DLD were obtained from school or medical files, or tested when unavailable (*n *= 8). Children without DLD were included in the study when their PIQ fell within the 95% Confidence Interval of a PIQ of 85. Performance IQ data were missing for four children (three of them with DLD).

The Child Alexithymia Measure (CAM, Way et al. 2010) was used to assess the children’s difficulty in communicating about their emotions. Parents indicated how often their child avoids emotional topics, has difficulty expressing emotions, or shows incongruent emotion expression and emotion communication (almost never (1), sometimes (2), often (3), almost always (4)). The questionnaire had good internal consistency in both groups (*α *> .90), as in the validation study (Way et al. 2010). Data were missing for 17 children with DLD (16.3%) and 32 children without DLD (17.5%) due to non-response of parents.

The severity of communication problems was examined with the parent-reported Children’s Communication Checklist (CCC-2-NL; Geurts et al. 2009; Norbury et al. 2004), which measures the severity of structural language problems (speech, syntactic, semantic, coherence problems) and pragmatic problems of children (initiation of conversations, non-verbal communication, use of context, and stereotypical language use). In addition, all scales of the CCC-2-NL can be summed providing a general communication problems score (Norbury et al. 2004). Standard scores are available for the Dutch population. The pragmatic problems and general communication problems scales are reliable in both groups. However, the separate structural language scales are only reliable in children with communication disorders (Geurts et al. 2009) and were only examined for children with DLD. Data were missing for 16 (15.4%) children with DLD and 41 (22.4%) children without DLD due to non-response of parents, or because of unreliable answers in the positively stated questions.

When the parent questionnaires (CCC-2-NL and CAM) were used in the analyses, children for whom this data was missing were excluded. However, children without DLD whose parents did not fill out the parent’s questionnaires were older (*t* (182) = − 2.43, *p *= .016) and had lower PIQ (*t* (181) = 3.88, *p *< .001) compared to the children for whom all information was available. In the DLD group, no differences were found on any of the study variables. Additionally, the analysis revealed that the pattern of results was not different when children with missing data were excluded. Consequently, we were confident that the missing data did not affect our results.

### Study Procedure

Children filled out all self-report measures at two time points with a 9-month interval. Children were tested individually in a quiet room at school or at home. All questionnaires were presented on a laptop or tablet. For children with DLD, all questions were read aloud. It was explained that all the answers were to be treated anonymously and that there were no right or wrong answers. In addition, parents filled out questionnaires about general communication problems and emotion communication of their child at Time 1. Prior to participating, all parents and children above 12 years of age signed an informed consent form. The study was approved by the local Ethical Committee of Leiden University in the Netherlands.

### Statistical Analyses

Preliminary analysis showed that there were no differences between children with DLD in special education or in mainstream education so both groups were combined. There were also no differences on any of the study variables between children who participated on two time points and children who only participated once (with DLD = 14, without DLD = 27).

Because we have two data-points for the participants, we performed longitudinal analyses using multi-level modeling in R (R Core Team 2016), which uses all the available data of participants, and models the dependency within the data (Singer and Willett 2009). We used Maximum likelihood estimation in the analyses, assuming that the missing data were missing at random.

We compared the model fit of increasingly more complex models. Models were preferred when they accounted for the most variance within the data with the fewest number of predictor variables. Model fit was compared with the Akaike information criterion (AIC). Models were only reported when the AIC was significantly lower with *p *< .05 (Singer and Willett 2009). The distribution of social anxiety and somatic complaints were positively skewed in both groups. Therefore, we used a clustered bootstrap procedure with 5000 samples as a robust method to deal with non-normally distributed data (Field 2017). Predictors are significant when 0 is not in the 95% Confidence Interval (CI). Assumptions of linearity, multicollinearity and homoscedasticity were met. Additionally, residuals of the final models were normally distributed.

We first examined the level and development of social anxiety, somatic complaints, emotion awareness and happiness in both groups, while controlling for age, gender, PIQ and SES. Second, we examined the contribution of happiness and emotion awareness to the prediction of social anxiety and somatic complaints. Third, we examined the relations between social anxiety and somatic complaints with the general communication problems and the emotion communication problems of children with and without DLD. Because these communication variables were only measured once, we were unable to perform longitudinal analyses with these variables.

## Results

### Group Differences and Developmental Trajectories

The first aim of the study was to examine the level and development of somatic complaints, social anxiety, happiness, and emotion awareness in both groups. We fitted a basic means model as baseline and added age (centered around the mean) as a time-varying predictor. The control variables SES, gender, and PIQ were added one by one. The control variables were only kept in the model when they made a significant contribution to the model. PIQ did not contribute to any of the models, nor did the contribution of PIQ change the pattern of results of other factors. Therefore, PIQ was excluded from all analyses. Next, diagnosis (without DLD = 0, with DLD = 1) and the interaction between diagnosis and age were added to the model in order to examine differences in the level and development of somatic complaints, social anxiety, happiness, and emotion awareness across time between groups.

**Fig. 1:**
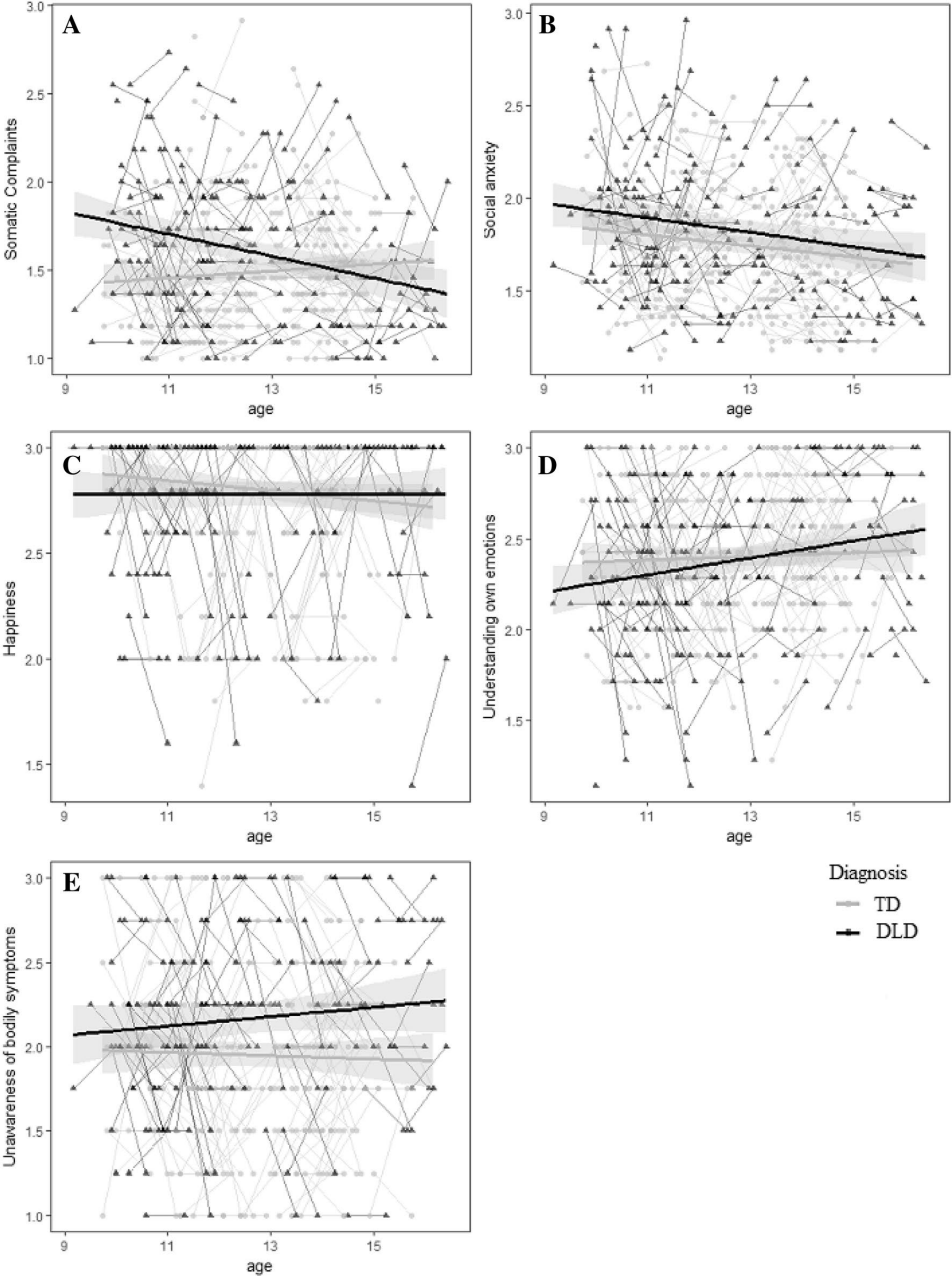
Representation of the raw data for somatic complaints (**a**), social anxiety (**b**), happiness (**c**), emotion understanding (**d**), and bodily unawareness of emotions (**e**). The regression lines represent the mean level in children with and without DLD across time with 95% CI

The best fitting models are presented in Table 3 (see the Supplementary Material for model fit indices). On mean levels, children with DLD reported more somatic complaints and social anxiety than children without DLD. In children with DLD, both internalizing problems decreased as children became older. In children without DLD only social anxiety decreased across time, whereas somatic complaints increased in older children without DLD. Positive emotions and emotion understanding did not differ in children with or without DLD. Emotion understanding increased as children became older. Children with DLD reported less awareness of bodily symptoms in response to emotions (or more external focus) than children without DLD. As Fig. 1 shows, there were many individual differences across time.

**Table 3:** Regression weights with 95% CI for the best fitting models

	Somatic complaints	Social anxiety	Happiness	Emotion understanding	Bodily unawareness
Age	**.019** [.002, .051]	− **.031** [− .058, − .007]	− .012 [− .035, .007]	**.028** [.011, .061]	.009 [− .015, .049]
SES	− .045 [− .087, .006]	–	–	–	–
Gender	**.079** [.005, .161]	–	–	–	− **.153** [− .273, − .049]
Diagnosis	**.093** [.002, .194]	**.105** [.012, .192]	–	–	**.201** [.093, .320]
Diagnosis × age	− **.080** [− .131, − .046]	–	–	–	–

Significant regression weights are bold. Gender: girls = 1; Diagnosis: DLD = 1

## The Role of Happiness and Emotion Awareness in Somatic Complaints and Social Anxiety

The second aim of the study was to examine happiness and emotion awareness as protective factors for the level of social anxiety and somatic complaints. Therefore, we examined whether differences between persons in somatic complaints and social anxiety could be explained by the level of happiness and emotion awareness, and whether growing levels of happiness or emotion awareness within persons were longitudinally related to decreasing levels of social anxiety and somatic complaints. We first computed the mean happiness and emotion awareness on both time points for individual participants. This mean score was added to the model to examine whether between-person differences in happiness or emotion awareness explained differences in the level of somatic complaints or social anxiety. We then computed person specific change scores (score at both time points minus the mean score) for happiness and emotion awareness. The change scores were added to the model to examine whether within-person changes in happiness and emotion awareness were longitudinally related to changes in the level of somatic complaints and social anxiety across time (Singer and Willet 2009). In order to examine whether the relations were moderated by DLD, the interaction terms of diagnosis and Mean and Change of the happiness and emotion awareness were added to the model.

As Table 4 shows, higher mean levels of happiness, emotion understanding, and bodily unawareness, as well as increasing levels of emotion understanding across time (change) were related to lower levels of somatic complaints and social anxiety in both groups. For children with DLD, the relation between mean bodily unawareness and somatic complaints was stronger. Additionally, in children with DLD only, increasing levels of bodily unawareness (change) were related to decreasing social anxiety. The interaction effects of diagnosis × emotion understanding and diagnosis × happiness did not contribute to the model and were excluded.

**Table 4:** Regression weights with 95% CI for best fitting models with emotion awareness and happiness predicting somatic complaints and social anxiety

	Somatic complaints	Social anxiety
Age	.014 [− .004, .040]	− .021 [− .039, .003]
Neighborhood SES	− .030 [− .061, .011]	–
Gender	.030 [− .029, .096]	–
Diagnosis	**.527** [.241, .826]	.342 [− .047, .672]
Diagnosis × age	− **.050** [− .087, − .017]	–
Happiness		
Mean	− **.381** [− .505, − .265]	− **.252** [− .376, − .133]
Change	− .066 [− 187, .052]	.003 [− .135, .131]
Emotion understanding		
Mean	− **.228** [− .307, − .126]	− **.338** [− .460, − .221]
Change	− **.126** [− .231, − .025]	− **.154** [− .285, − .020]
Bodily unawareness		
Mean	− **.135** [− .207, − .057]	− **.198** [− .296, − .115]
Change	− .026 [− .112, .061]	.011 [− .101, .123]
Diagnosis × bodily unawareness		
Mean	− **.191** [− .320, − .064]	− .096 [− .244, .079]
Change	− .097 [− .275, .097]	− **.318** [− .538, − .087]

Significant regression weights are bold

## The Role of (Emotion) Communication in Somatic Complaints and Social Anxiety

The third aim of the study was to examine whether general communication skills and the ability to talk about emotions were related to the severity of social anxiety and somatic complaints, also in addition to emotion awareness and happiness. First, Pearson’s correlations between the communication problems and the other study variables were examined (Table 5). For children with DLD, we also examined the structural language scales (speech, syntax, semantics, and coherence). Second, the models with the control variables were rerun excluding children with missing data on the (emotion) communication questionnaires. Third, either emotion communication, pragmatic problems, or the GCS as well as the interaction effects with diagnosis were added to the model. Fourth, the contribution to the models of the separate communication problem scales were examined for children with DLD alone. Finally, in order to examine the unique contribution of the different predictors, happiness and emotion awareness were added to the models.

**Table 5:** Pearson’s correlations with the communication problem scales in children with and without DLD

	With DLD							Without DLD		
	Speech	Syntax	Semantics	Coherence	Pragmatics	General	Emotion	Pragmatics	General	Emotion
Somatic complaints	.22*	.06	.16	.16	.17	.21*	.26*	− .07	− .06	.09
Social anxiety	.21*	.08	.18	.17	.25*	.26*	.10	− .05	− .11	.09
Happiness	.02	.03	− .02	.04	− .09	− .03	− .22*	.05	.04	.03
Emotion understanding	− .22*	− .09	− .21*	− .29*	− .18	− .25*	− .10	.21*	.18*	− .12
Bodily unawareness	− .15	− .02	− .23*	− .18	− .10	− .16	− .08	.24*	.28**	− .02
Emotion communication	.10	.14	.15	.12	.41***	.32**	–	.20*	.14	–

*p < .05; **p < .01; ***p < .001

The results showed that higher levels of general communication problems and pragmatic problems were unrelated to somatic complaints in both groups when the control variables were taken into account. Additionally, none of the communication scales (speech, syntax, semantics, coherence, pragmatics and general communication problems) were related to the level of somatic complaints when only the children with DLD were examined. In contrast, emotion communication problems were related to more somatic complaints in both groups (*B *= .117 [.032, .200], also in addition to emotion awareness and happiness (*B *= .073 [.001, .147]). The contribution of emotion awareness and happiness to both internalizing problems remained when emotion communication was controlled for.

More general communication problems were related to more social anxiety, but only in children with DLD (*B *= .006 [.003, .017]). Pragmatic problems were unrelated to social anxiety when both groups were examined together. However, when only children with DLD were taken into account, more pragmatic problems were related to higher levels of social anxiety (*B *= .001 [.011, .022]). Emotion communication was unrelated to social anxiety in both groups. Additionally, none of the structural language scales contributed to social anxiety in children with DLD.

As Table 5 shows, more structural language problems in children with DLD (speech, semantics and coherence) were related to lower levels of emotion understanding and more semantic problems related to less bodily unawareness. When emotion understanding, bodily unawareness and happiness were added to the model predicting social anxiety, the general communication problems and pragmatic problems no longer contributed to the model.

## Discussion

Since individuals with DLD are at risk of developing internalizing problems which can have a strong, negative impact on people’s lives, it is important to gain insight into which factors could be addressed in interventions in order to prevent suboptimal developmental trajectories. The present study identified for the first time several protective factors for the development of social anxiety and somatic complaints in DLD. This is of particular relevance, since the communication problems seem to be stubborn and persistent (Norbury and Sonuga-Barke 2017), challenging the potential success of interventions (McCartney 2017).

According to our longitudinal study, having higher levels of positive emotions, being aware of the causes and consequences of emotions, and focusing less on internal bodily states of emotions were linked to lower levels of social anxiety and somatic complaints in children with and without DLD. Additionally, growing awareness of emotions was linked to decreasing social anxiety and somatic complaints. These findings suggest that emotion awareness may have a protective function for the development of internalizing symptoms in children with and without DLD.

In addition to the protective functioning of emotion awareness and happiness for the development of internalizing problems, the current study also examined the relations with (emotion) communication abilities. Difficulties expressing oneself may create misunderstandings and frustration resulting in more stress related to somatic complaints or anxiety in social interactions. However, it could also be that the general language problems are not the main problem. Instead, the communication problems may cause an inability to effectively differentiate and communicate emotions, which in turn puts children at risk for internalizing problems. As in previous studies, we found mixed results between the severity of communication problems in children with DLD and their internalizing problems (St Clair et al. 2010; Van Daal et al. 2007).

For somatic complaints, no relation was found between the structural and pragmatic language problems after controlling children’s age, gender and SES. However, somatic problems were higher in children with and without DLD who experienced problems to communicate about their own emotions according to their parents and who reported to be less aware of their own emotions. This suggests that it is not the communication problems themselves, but rather the inability to communicate and differentiate emotions that is an important area to focus on in interventions. When children are unable to understand what they are feeling and why, it is more difficult to deal with the cause and consequences of an emotional situation. Additionally, when children are unable to express their emotions, other people are less able to support them in their emotional experiences, to help them regulate their emotions, for example. Moreover, when children are unable to explain to others what they are feeling and why, it is more likely that the situation will remain unchanged, potentially fueling increasing levels of the (negative) emotional experience (Eisenberg et al. 2005; Gross 1998, 2015). As a consequence, the emotional experience of children may remain high, causing stress reactions in the body such as tensed muscles, which may lead to increased somatic complaints. Therefore, it is important to help children understand what they are feeling, what causes their emotions, and how they can constructively react to their emotions.

For social anxiety, a different pattern of results was found. Emotion communication problems were unrelated to the level of social anxiety in children with and without DLD. The severity of general communication problems (the sum of structural and pragmatic problems) and the pragmatic problems was related to more social anxiety in children with DLD. However, the severity of communication problems was no longer related to social anxiety when emotion awareness was taken into account. This suggests that difficulties understanding emotions rather than the severity of communication problems causes stress reactions in social interactions in children with DLD. Therefore, it is important to help children understand their own emotional experiences and recognize the causes of these emotions.

Interestingly, children with DLD with more severe structural language problems reported less emotion awareness. This suggests that the communication difficulties of children with DLD have a negative impact on the development of emotion awareness which in turn puts them at risk for internalizing problems. A previous study found that use of this causal emotion language is especially important for better emotion understanding in children with DLD (Yuill and Little 2018). Therefore, caregivers and professionals can help children, not only by labelling different emotions, but also by explaining the causes and consequences of emotions.

The findings of the current study suggest that prevention and intervention programs focusing on increasing emotion awareness may be beneficial for individuals with DLD to the same extent as for children without DLD. Focusing on language improvement, which has been shown to have only transient or limited effects (McCartney 2017), combined with increasing emotion awareness may be more promising, since emotional competences may be more malleable.

Interestingly, it appears that happiness and emotion awareness seem to have comparable effects in both groups, in spite of the reports of higher levels of somatic complaints and social anxiety in the DLD group. There were a few exceptions, however, which may suggest an intervention specifically tailored to DLD would be more effective. For example, compared to children without DLD, children with DLD reported more somatic complaints and lower bodily awareness when emotional. Moreover, decreasing levels of bodily awareness were related to decreasing social anxiety in children with DLD only. Although these results should be interpreted with caution since the internal consistency of the bodily awareness scale was rather low, the results suggest that children with DLD who focus on the causes of their emotions instead of having an inward focus are less vulnerable to developing internalizing problems. This, in turn, may be a good starting point for intervention or prevention programs.

Although the content of interventions could be similar for children with and without DLD, special care should be taken to make interventions accessible for children with DLD. Firstly, the amount and level of language in an intervention should be adapted to the language abilities of the child. Secondly, visual material should be used to facilitate the understanding and discussion of emotional situations. Preferably, ecologically valid material should be used such as pictures and videos of interactions of children. These materials could be used to discuss the thoughts, emotions and resulting behavior of multiple people in particular situations. By making the implicit thoughts and feelings explicit, children will gain better insight concerning their own and others’ emotions and gain the necessary emotion language to reflect upon and discuss emotions (Brinton and Fujiki 2011; Dunn et al. 1991). Finally, it is important to gain understanding of the level of emotional awareness which is needed to understand the intervention. When said basic abilities are absent, it is likely that children would benefit less from such interventions.

### Limitations

The present study is one of the first studies to look at protective factors that are linked to internalizing symptoms in individuals with DLD. However, there are a few limitations of the current study. For several measures (communication difficulties and difficulties to communicate emotions) we only had data from one time point, which prevented us from examining whether changes in these skills contributed to the development of internalizing problems. Additionally, we primarily used child-report questionnaires which could provide common method bias. Yet, for the emotion communication only parent reports were used. Future studies would benefit from using multiple informants to examine whether children, parents and peers experience, perceive and report problems to the same extent. Furthermore, the age range of our participants was quite large, whereas the mechanisms underlying internalizing problems may change as children develop into adolescence. Power issues prevented us from examining age-related differences more closely, which would be an interesting topic for future studies. Finally, longitudinal studies in younger age groups would help to gain a better understanding of causal effects of emotion communication problems on the development of internalizing problems in children.

## Conclusions and Outlook

We were able to identify several protective factors for internalizing symptoms in individuals with DLD. This has implications for future prevention or intervention programs aiming to reduce internalizing symptoms in individuals with DLD. Our study suggests that such programs may benefit from focusing on increasing positive emotions (Quoidbach and Gross 2015), emotion understanding and bodily unawareness (Havighurst et al. 2010; Suveg et al. 2006; Wilamowska et al. 2010). Moreover, being better able to communicate about emotions may help reduce internalizing symptoms in the long run (Brinton and Fujiki 2011; Brumariu and Kerns 2015; Mathews et al. 2016).

## Electronic supplementary material

Below is the link to the electronic supplementary material.

Supplementary material 1 (DOCX 37 kb)
