# Supplementary material for: Positive Aspects of Emotional Competence in Preventing Internalizing Symptoms in Children with and without Developmental Language Disorder: A Longitudinal Approach
Source: J Autism Dev Disord. 2020 Jan 6;50(4):1159–71. doi: 10.1007/s10803-019-04336-y (PMC7101296; doi:10.1007/s10803-019-04336-y)
Supplement: Supplementary file 1 — Supplementary material 1 (DOCX 37 kb) [file 10803_2019_4336_MOESM1_ESM.docx]

**Supplementary Material**

Goodness of fit (AIC and log likelihood test) of the different models

| Model | Somatic  complaints | Social  Anxiety | Happiness | Emotion  Understanding | Bodily Unawareness |
| --- | --- | --- | --- | --- | --- |
| Basic means model | 342.89 | 467.55 | 334.30 | 506.34 | 755.33 |
| Addition of: |  |  |  |  |  |
| Age | 341.76 | 462.71** | 334.89 | 503.11* | 757.18 |
| Gender | 339.82* | 463.05 | 336.45 | 504.55 | 750.01** |
| SES | 333.28** | 462.43 | 333.55 | 504.79 | 757.95 |
| Diagnosis | 330.28* | 459.17* | 336.34 | 504.55 | 740.01*** |
| Diagnosis*age | 318.79*** | 460.32 | 336.89 | 504.63 | 741.10 |
| Happiness, emotion understanding,  bodily unawareness | 172.72*** | 318.39*** | - | - | - |
| Diagnosis x Bodily unawareness | 167.16** | 310.25** | - | - | - |

*Note.* **p*<.05, ***p*<.01, ****p*<.001
